# Antiviral effect of cannabidiol on K18‐hACE2 transgenic mice infected with SARS‐CoV‐2

**DOI:** 10.1111/jcmm.70030

**Published:** 2024-09-12

**Authors:** Hivda Ulbegi Polat, Hicret Asli Yalcin, Deniz Köm, Özge Aksoy, Irem Abaci, Arzu Tas Ekiz, Müge Serhatli, Selma Onarici

**Affiliations:** ^1^ TUBITAK Marmara Research Center, Life Sciences, TUBITAK Kocaeli Turkey; ^2^ Molecular Biology and Genetics, Institute of Sciences Yildiz Technical University Istanbul Turkey; ^3^ Department of Biotechnology, Institute of Biotechnology Gebze Technical University Kocaeli Turkey

**Keywords:** antiviral, cannabinoid, cannabis, CBD, COVID‐19, mice, SARS‐CoV‐2

## Abstract

The aim of this study was to determine the antiviral activity of cannabidiol (CBD) against SARS‐CoV‐2 infection. CBD is the second most studied cannabinoid obtained from *Cannabis* plants. We investigated the potential use of CBD, which has so far proven to have a positive effect on different diseases, in the SARS‐CoV‐2 infection. To test this, in vivo studies were carried out using K18‐hACE2 transgenic mice. To reveal the potential therapeutic effect of the CBD at the histopathological and molecular level challenge experiments were performed. The study was designed with two groups (*n* = 10) and in the treatment group animals were infected with SARS‐CoV‐2 virus strain B.1.1.7 alpha before the administration of CBD. While the disease progressed and resulted in death in the control group that was infected by the virus alone, it was observed that the infection slowed down and the survival rate increased in the mice treated with CBD along with the virus. In this study, K18‐hACE2 transgenic mice infected with the wild SARS‐CoV‐2 virus were used to investigate and prove the antiviral activity of CBD.

## 1 INTRODUCTION

Coronavirus disease 2019 (COVID‐19), a respiratory infectious disease caused by severe acute respiratory syndrome coronavirus 2 (SARS‐CoV‐2), has been impacting the entire world since 2019.^1^ The World Health Organization (WHO) then declared COVID‐19 disease as global pandemic in March 2020.^2^, ^3^ The virus is similar to the genomes of SARS‐CoV, which appeared in 2002, and MERS‐CoV, which broke out in 2012, with 79% and 50% homology, respectively.

The presence of inflammatory responses followed by acute respiratory distress syndrome (ARDS) in COVID‐19 results in significant implications, such as cytokine storm.^4^ Studies have been conducted to investigate the administration of traditional or herbal medications, either individually or in combination, to COVID‐19 patients with the aim of preventing the entry, replication and transmission of the virus.^5^, ^6^, ^7^, ^8^ The use of herbs can help the immune system by providing antiviral, antioxidant, anti‐inflammatory and immune‐boosting properties, which can eventually reduce infection‐related mortality. These characteristics make them suitable candidates for COVID‐19 treatment.^9^

*Cannabis* is a genus of flowering dicotyledonous plants in the Cannabaceae family. Because of their high oil and fibre content, *C. sativa* and *C. indica*, both members of the Cannabaceae family, are produced and used in industrial applications.^10^ However, they are most commonly utilized as herbal medicines due to their secondary metabolite content, which includes cannabinoids, flavonoids, steroids and terpenoids. Cannabinoids, specifically tetrahydrocannabinol and cannabidiol (CBD), have received the most attention because of their potential applications in the treatment of epileptic seizures, nausea, anxiety disorders, chronic pain and inflammatory diseases.^11^, ^12^, ^13^

After several critical research studies on the possible therapeutic effects of CBD, the US Food and Drug Administration (FDA) has given approval to CBD‐containing drugs. First, Epidiolex^14^ was approved by the FDA in 2018 for the treatment of epileptic seizures related to Lennox–Gastaut syndrome and Dravet syndrome.^15^, ^16^ Second, the same drugs were approved by the FDA in 2020 for the treatment of tuberous sclerosis complex (TSC)‐related seizures.^17^, ^18^

While the potential is increasing, little research has been conducted to investigate the potential therapeutic effects of CBD on COVID‐19 or the clinical symptoms caused by SARS‐CoV‐2.

By using in vivo tests, we aimed to establish the efficacy of the CBD molecule against upper respiratory tract viral infections. Our study was conducted to establish the antiviral impact of the CBD molecule, which had not before been demonstrated. All researchers should acknowledge the difficulties of dealing directly with a pandemic virus. We used an intranasal injection of the SARS‐CoV‐2 virus to directly infect model organisms, examining the potential therapeutic effects of CBD. This study is important because it tested various doses of CBD on mice infected with the SARS‐CoV‐2 virus to determine its protective and therapeutic effects against upper respiratory tract infections. We attempted to establish the bioactivity of a plant‐derived CBD therapeutic candidate employing the SARS‐CoV‐2 virus in our investigation using a dosage study, acute toxicity and histopathology on K18‐hACE2 transgenic mice.

## 2 MATERIALS AND METHODS

### 2.1 Administration of CBD

One gram of CBD in crystal form with a minimum purity of 99% was purchased from Medicanna (Netherlands). Because CBD is a lipophilic chemical, multiple oils were utilized as solvents in this study, including olive oil and corn oil. Crystalline CBD was added in respective oil (olive oil with pH = 5, corn oil with pH = 7) with the concentration adjusted to 5 mg/mL. The mix was incubated at 50°C for 15 min and the mixture was vortexed in every 2–3 min. The mixture was stored at room temperature during the treatment of the animals.

### 2.2 Ethics statement and mice

The Jackson Laboratory in the United States provided the K18‐hACE2 [B6.Cg‐Tg (K18‐hACE2) 2Prlmn/J] transgenic mice were used in this study. TUBITAK Marmara Research Center (MRC), Life Sciences, Medical Biotechnology Unit in Gebze, Kocaeli, TÜRKİYE, approved 8‐ to 10‐week‐old male K18‐hACE2 transgenic mice. All procedures in this study involving animals were reviewed and approved by the Institutional Biosafety Committee and Institutional Animal Care and Use Committee (HADYEK‐16563500‐111‐6480); all the experiments were conducted in compliance with all relevant ethical regulations. The experiments were conducted in Biosafety Level 3 (BSL3) and animal BSL3 facilities at TUBITAK MRC Life Sciences.

### 2.3 Acute oral toxicity and dose study for CBD

To determine the effective viral dose on mice, LD_50_ criteria was used in this study. When more than 50% survival was achieved in intraperitoneal doses administered in the challenge test groups, higher doses were applied. In each group, the same amount of CBD was given to animals of the same weight living in the same environment.^8^, ^19^, ^20^

As this product is planned to be developed as oral supplement, the following challenge experiment was designed accordingly. To test acute oral toxicity on three different groups (*n* = 4) of K18‐hACE2 transgenic mice, the OECD standard 423^21^ was used. At the beginning, to test the effect of different oils, olive oil and corn oil were used. Initial concentration of CBD was 1 μg in 200 μL oil. *Group 1* received only olive oil, *Group 2* received only corn oil and *Group 3* received 1 μg of CBD dissolved in olive oil via intraperitoneal injection. According to the standards, each mouse received a single dosage of gavage, administered orally, with dose of 2000 mg/kg per mouse. Based on the results from gross pathology, none of the mice showed signs of toxicity or death at the end of the 7th day.^8^

To detect the antiviral activity of the CBD, after the standard treatment of each experimental group of transgenic mice with the SARS‐CoV‐2 virus, the first study was carried out with two experimental groups. The treatment group (*n* = 6) received 1 μg CBD dissolved in 200 μL olive oil per mouse and the control group (*n* = 6) only received olive oil.

In the following challenge study, different types of oils were used to test the effect of different oils on the antiviral activity of CBD molecules on the SARS‐CoV‐2 virus. We evaluated the second challenge experiment using olive oil and corn oil because corn oil is frequently utilized in animal research.^22^ To lower the toxicity of the oils, the amount of oil was reduced to 50 μL. One microgram of CBD per mouse was dissolved in 50 μL oil and 150 μL PBS at a dose of 2000 mg/kg per mouse. The experiment was designed to be conducted with three groups. Following SARS‐CoV‐2 virus treatment in all experimental groups, *Group 1* (*n* = 6) got 1 μg of CBD dissolved in corn oil. *Group 2* (*n* = 6) received 1 μg of CBD molecule dissolved in olive oil. And to *Group 3* (*control*) (*n* = 6) only 50 μL olive oil was given instead of CBD.

As the antiviral effect of CBD was positively observed in the second challenge study, the next challenge study was designed to test the effect of increased amount of CBD. The study was designed with two groups (*n* = 10), one for treatment and the other for control. Each mouse in the treatment group received 3 μg CBD dissolved in 50 μL oil and supplemented with PBS at a dose of 2000 mg/kg per mouse.

### 2.4 Intranasal delivery for SARS‐CoV‐2 infection in mouse models

**FIGURE 1:**
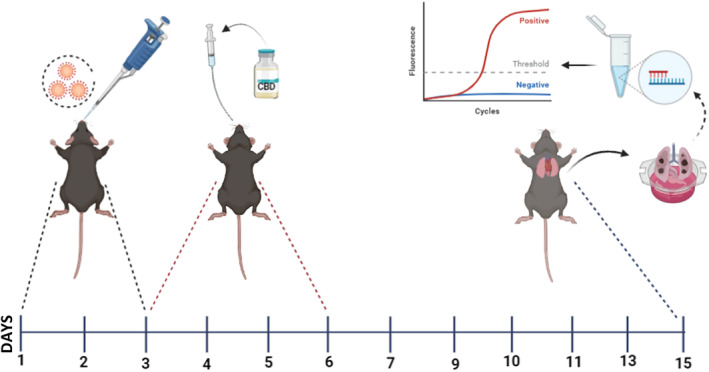
Schematic representation of in vivo experiment to test efficacy of cannabidiol (CBD) in vivo. K18‐hACE2 transgenic mice that express human ACE2 (hACE2) were infected with SARS‐CoV‐2 (Alpha). CBD medication, which began before infection, was continued during the viral administration (*n* = 10/group). On the 15th day of the experiment, lungs were taken and viral extraction was carried out. The viral titre was determined using RT‐PCR.

This study was designed to measure the therapeutic effect of the CBD molecule on SARS‐CoV‐2 virus. The Ministry of Health Public Health Directorate supplied the SARS‐CoV‐2 virus strain B.1.1.7 alpha. For the challenge study, the transgenic mice groups were taken to the BSL 3 laboratory 1 day prior to infection. The B.1.1.7 strain of the SARS‐CoV‐2 virus with a TCID_50_ value of 10^5^ was used in this study. Under anaesthesia, the SARS‐CoV‐2 virus was administered intranasally to the transgenic mice for 3 days.^23^ The first dose of the CBD molecule was started in the afternoon on the third day of infection, and the intraperitoneal injection was continued for a total of 4 days.^24^ The schematic explanation of the experimental plan can be seen in Figure 1. Each challenge experiment lasted a total of 15 days.

Following the SARS‐CoV‐2 viral infection, mice in each group were evaluated daily for morbidity (body weight) and mortality. Mice that lost more than 25% of their baseline body weight were deemed to have attained the experimental endpoint and were slaughtered. Pathological exams were carried out after the animals were killed. All abdominal and thoracic cavity organs were inspected with the naked eye during gross pathology. Organs that are clean and have no lesions are scored as 0, organs with oedema and hyperaemia are scored between 0.5 and 1, and organs with different rates of pneumonia lesions are scored between 1.5 and 5.

### 2.5 Histopathological analysis

For histopathological study, tissue samples were stored in 10% buffered formalin, dehydrated in a series of alcohol solutions of ascending concentration, and embedded in paraffin wax. Sections were cut to 5‐μm thick pieces which were stained with haematoxylin for 5 min and then with eosin. The stained sections were dehydrated, cleared and mounted to the slides. The slides were viewed under the Zeiss Axio Vert A1 microscope and Zen software 2.6 pro version (Zeiss, Oberkochen, Germany). Semi‐quantitative assessment was used in the study. The inflammation status in the lungs was compared to the whole lung and given a score between 0 and 3. Accordingly, 0 = no inflammation; 1 = morphologically normal but mild erythrocyte and lymphocyte infiltration around the bronchioles; 2 = moderate erythrocyte and lymphocyte infiltration in the lung; 3 = impaired morphology, intense erythrocyte and lymphocyte infiltration in the lung.

### 2.6 Isolation of viruses and RNA extraction for RT‐PCR

For real‐time PCR analysis, half of the mice's lungs were taken. Lung tissues were homogenized in 2 mL of PBS at 70% amplitude for 90 s using an ultrasonic homogenizer (Bandelin HD2200.2, Germany) for virus isolation. Tissue homogenates were centrifuged for 10 min at 21.500× *g*, and supernatants were collected. Viral RNA was isolated using the QIAamp Viral RNA Mini kit (QIAGEN, USA) according to the manufacturer's procedure. The viral RNA was detected using the One Step PrimeScript III RT‐PCR Kit (Takara, Japan) and SARS‐CoV‐2 nucleocapsid‐specific primers and probes described below. All reactions were carried out on a BioRad CFX96 Touch instrument (USA). The RT‐PCR protocol and primer‐probe information used in this study have been previously described.^8^

### 2.7 Statistical analysis

An unpaired Student's *t*‐test and two‐way ANOVA were used for comparison between the treatment and control groups. The data were presented as mean ± standard deviation. A two‐sided *p* value <0.05 was considered statistically significant. Statistical analyses and graphs were performed with GraphPad Prism 5 programs.

## 3 RESULTS

### 3.1 Challenge experiment for the determination of the doses

During the experiment, animals were studied after being given the SARS‐CoV‐2 virus at a TCID_50_ value of 10^5^ for 3 days. Body weight reduction defined the first clinical symptoms in animals.

In the first challenge experiment, it was observed that two of six mice from the CBD‐treated group that received 1 μg CBD dissolved in 200 μL olive oil lost 13% and 15% of their body weight, respectively. The remaining four mice (~66%) were observed to keep their good health conditions until the end of the experiment. However, in the control group, which received only olive oil, between 16% and 25% weight loss was recorded for all animals. When harvested lungs were evaluated for gross pathology, the lungs of one mouse from the CBD group had hyperaemia. However, in the control group, all six mice were observed to have pathological lesions at different rates, ranging from 1 to 5. The CT values, which were used to determine viral load on the targeted tissue, were also found to be consistent with the clinical and gross pathology results. In RT‐PCR results from the two mice that lost weight in the CBD treatment group (*n* = 6), low CT values (CT <20), indicating a high viral load, were detected. A high viral load was also detected in all mice in the control group (*n* = 6), with CT values changing between 13 and 18 (Table S1).

The second challenge experiment was conducted with different types of oils to detect if there was any effect of them on the antiviral activity of CBD treatment. In *Group 1* (*n* = 6), which received 1 μg CBD dissolved in corn oil, three mice lost around 10%–25% of their body weight, while two experienced a body weight loss of around 3% and 6%, respectively. Besides that, in *Group 2* (*n* = 6), which received 1 μg CBD dissolved in olive oil, animals were less affected by the infection, and only three animals lost 6%–13% of their body weight. However, all the animals in *Group 3* (*n* = 6) (control) were observed to have lost 6%–44% of their body weight. Different aspects are evaluated in gross pathology, like tissue integrity, organ appearance and the size of the organs. Mouse lungs from CBD‐treated groups were observed as clear; however, the lungs of the control group had different rates of pathological lesions. According to the RT‐PCR results of *Group 1 of Study 2*, low CT (CT <29) indicating a high viral load was found in four of six mice. However, in *Group 2* of *Study 2* only one mouse showed low CT value (CT = 26) and for the rest (~83%), the viral load was too low to be detected. When *Group 3* of *Study 2* (control group) was evaluated, five of the six mice had a high viral load, with CT values changing between 18 and 25, and only for one animal the viral load was not detected (Table S1).

**FIGURE 2:**
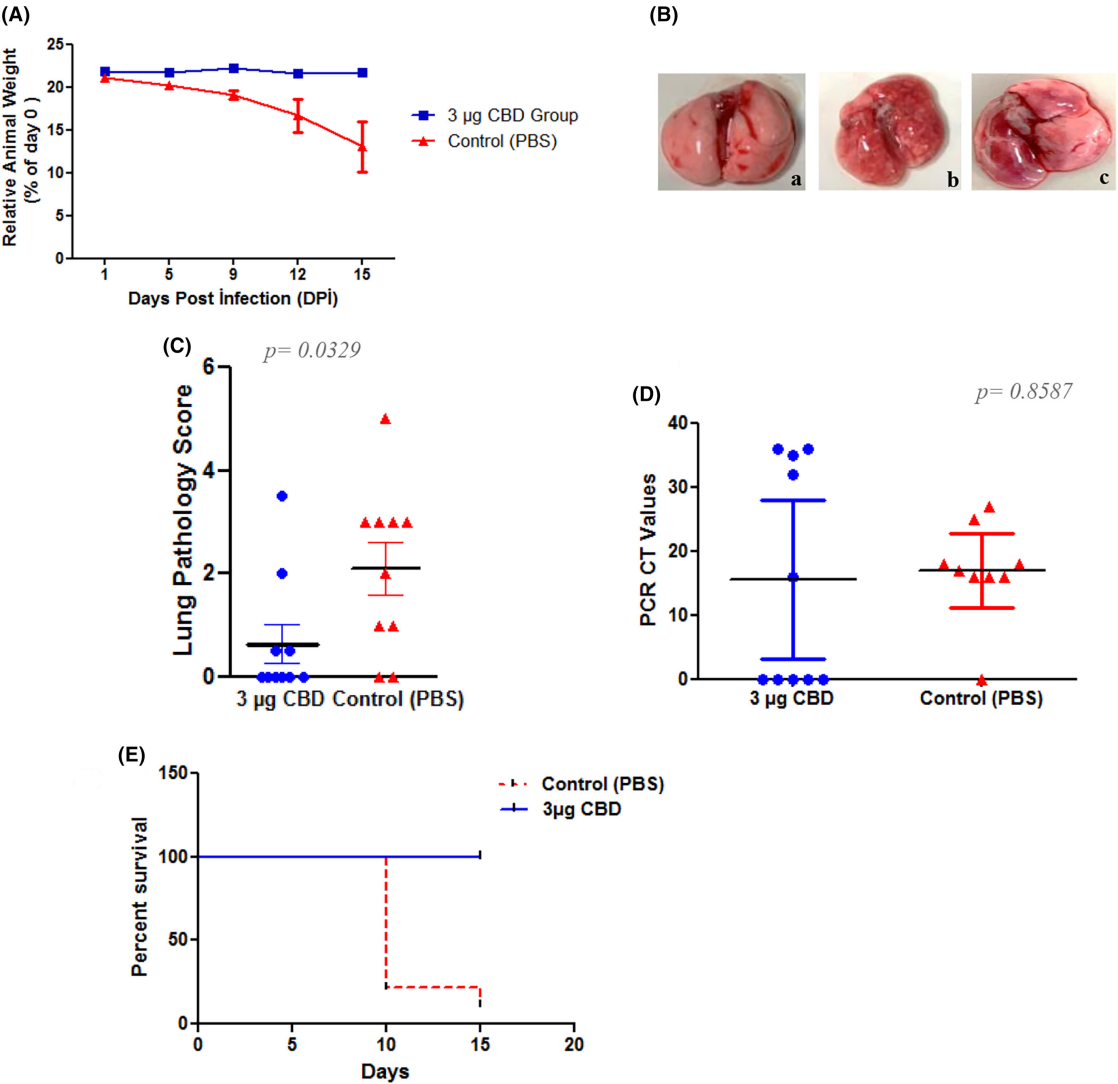
Neutralization effect of cannabidiol (CBD) drug in vivo. (A) Average change in body weight by time in control and CBD‐treated mice. Only 12th and 15th days were significant (***p = 0.0001 for both days) between the groups. A two‐way ANOVA was applied for statistic. (B) Representative lung images dissected from control and CBD‐treated mice. ***a*:** Treatment of lungs with 3 μg CBD drugs. ***b*:** Lung infection onset (control group). ***c*:** Lung infection with pneumonia (control group). (C) Gross pathology inflammation scores from control and CBD‐treated mice lungs. Lung inflammation was scored from 0 to 3 (0: No inflammation, 1: Low, 2: Medium, 3: High). Results for the gross pathology in the lung were significant (*p = 0.0329). (D) Viral loads in lungs from control and CBD‐treated mice (*n* = 10), measured via real‐time PCR targeting two different regions of SARS‐CoV‐2 nucleocapsid gene. Results for the viral loads in the lung were significant (*p = 0.08587). A *t*‐test was applied for (C, D) statistics. (E) The survival graph shows the animal survival rate (***p = 0.0001).

As showed in the results of the second study, mice given a dose of 1 μg of CBD in olive oil recovered from the disease, and most of the mice did not show even clinical symptoms. At the last study, the challenge experiment was continued with olive oil. But this time, to unleash the antiviral potential of the CBD molecule, the study was designed with a dose of 3 μg CBD/mouse, with 10 mice in each group. At the end of the experiment, in the CBD‐treated group, except for three animals that lost around 5%–9% of their body weight, 7 of the 10 mice showed no significant weight loss. In contrast to the treatment group, 7 mice in the control group had dramatic weight loss of between 23% and 33% of their body weight. These animals started to show severe clinical symptoms such as hunched posture, diarrhoea, bloody urine, burring in the eyes, adhesion due to this burr in some, and abdominal breathing on the 9th and the 10th days of the challenge experiment. On the 15th day, one animal from the control group was found dead in its cage. The weight chart of the groups is shown in Figure 2A.

Gross pathology scores were used to evaluate the pneumonia picture in the lung. The CBD‐treated group mice were found to be clean and showed no signs of infection in their lungs. All control animals were found to have pneumonia of varying sizes in their lungs and scored according to its degree (Figure 2B,C). To reveal the viral loads in harvested lungs RT‐PCR targeting the SARS‐CoV‐2 virus nucleocapsid gene was applied and the viral load of each sample is interpreted according to the CT values.^25^, ^26^ According to RT‐PCR results, in the control group, 8 of the 10 animals were detected to have a high viral load, with CT values ranging between 15 and 27. In 10 control mice, one of them died due to the infection, and the viral load was not detected in one animal due to individual differences. In contrast to the control group, no viral load was detected in 5 of the 10 animals treated with CBD. In four mice, a low viral load with a CT value ranging between 32 and 36, which would be considered insignificant, was detected. However, for one mouse, CDB did not provide healing, and a high viral load (CT value 16) was detected in the animal. Compared to control animals, it was shown that the experimental group's animals receiving CBD offered 90% more protection. Significantly different viral loads in the experimental groups could be seen graphically in Figure 2D.

**FIGURE 3:**
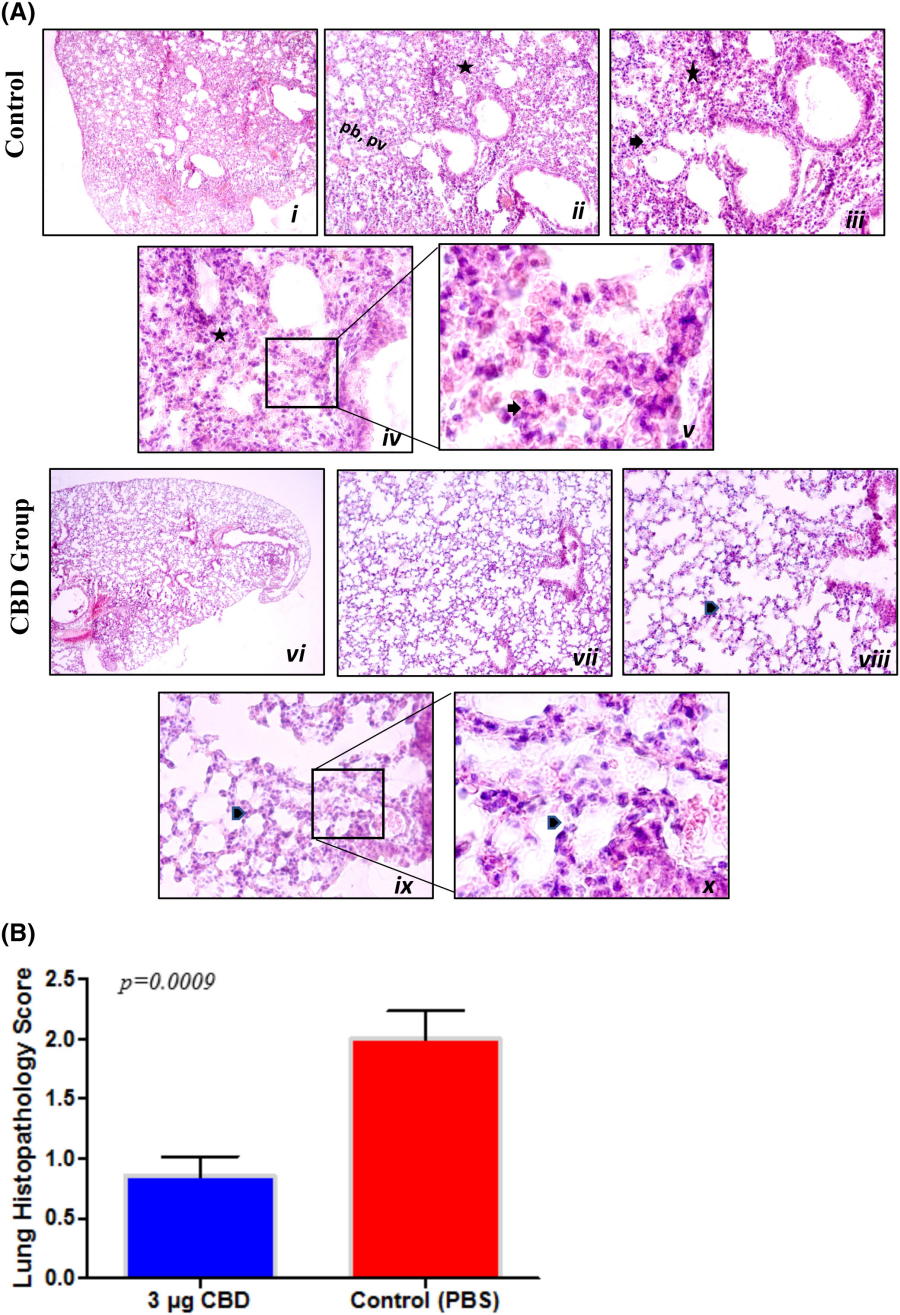
The IHC graph of all mice and several representative IHC images of lung tissue from the control (*i*) and cannabidiol (CBD) (*ii*) groups. (A) Representative IHC images of lung tissues obtained from control (*i–v*) and test (*vi–x*) groups. The tissue architecture of control group shows interstitial inflammatory cell infiltration (asterisk, *ii, iii* and *iv*), alveolar septal thickening (arrow, *iii* and *v*) and more parenchymal infiltration at peribronchiolar (pb, *ii*) and perivascular (pv, *ii*) regions. Test group shows decreased amount of interstitial inflammatory cell infiltration and normal alveolar morphology (arrowhead, *viii, ix* and *x*). Image magnification of *i* and *vi* are 4×, *ii* and *vii* are 10×, *iii* and *viii* are 20×, *iv* and *ix* are 40×, *v* and *x* are 100×. (B) Histopathology inflammation score graph of the lungs from the control and CBD group. Results for the inflammation score in the lung were significant (***p = 0.0009). A *t*‐test was applied for (B) statistics.

Following the staining procedure with haematoxylin and eosin, the lung sections were analysed, and lung inflammation was semi‐quantitatively scored between 0 and 3. When comparing the groups, the control group was detected to have significantly higher histopathology scores than the CBD‐treated group. The lungs of animals treated with CBD showed a healthy condition, whereas the control group showed pathological differentiation. Observed differences were in their interstitial inflammatory cell infiltration, alveolar septal thickening and more parenchymal infiltration in the peribronchiolar regions (Figure 3A).^23^, ^27^, ^28^ The histopathology inflammation score of the lungs from the control and CBD groups is illustrated in Figure 3B.

## 4 DISCUSSION

Since the emergence of COVID‐19 and its declaration as a pandemic, in vivo model studies with K18‐ACE2 transgenic mice against the disease have been started quickly, and this model is still actively being used today. In 2020, Winkler and his colleagues observed that after intranasal infection of K18‐ACE2 mice at 4dpi and 7dpi, the animals lost significant weight and had high viral loads at 2dpi, 4dpi and 7dpi.^2^ In the high viral load case once more investigated in the Bao and his colleagues' study, the authors detected the highest levels of virus in the lungs of hACE2 mice at 3dpi, then the load decreasing to 5dpi, and continuing to decrease to 7dpi.^29^ With the guidance of these studies, to establish CBD protection and prevent the development of full disease in our animals, an intranasal administration of B.1.1.7 virus with a TCID_50_ value was carried out daily for 3 days (Figure 1).

In the treatment and control groups, on the critical days of the infection (3dpi, 5dpi and 7dpi), the animals were clinically followed. Due to CBD is therapeutic, our challenge assay was created with its use in humans in consideration. The CBD molecule was deemed to provide excellent therapeutic effects against disease even administered orally 2 days after the infection.

There are studies revealing that herbal medicines have the feature of stopping virus replication by inhibiting the production of proinflammatory cytokines and reducing acidity in endosomes.^30^, ^31^ Chatow and his colleagues' study determined that the various CBD concentrations utilized had no effect on cell viability and were non‐toxic. In our work, we tested the acute toxicity of CBD in different solvent oils on mice. According to the CBD cell investigation, no mice died or showed symptoms of toxicity after day 7.^32^ Alongside the revealed potential usage as a preventative strategy, it has also been hypothesized that CBD could be used to limit disease progression due to its anti‐inflammatory and immunomodulatory effects.^33^ Various in vitro investigations have shown the antiviral activity and therapeutic effect of CBD against SARS‐CoV‐2 in this context.^32^, ^34^

In studies conducted by Khodadadi and Salles in 2020, ARDS‐like symptoms were induced by intranasal administration of Poly(I:C), a synthetic mismatched double‐stranded RNA created to reproduce the histopathological features of ARDS.^35^, ^36^ In the comparative analyses performed between the control group and the Poly(I:C)‐treated group, a reduction in apelin immunoreactivity was observed. This reduction was reversed after treatment with CBD and the apelin expression was also observed to increase towards control levels in the lung tissue. Besides that, the pathological features due to polyI:C administration were completely or partially lost by following treatment with CBD.^36^ In our study, we used a mouse model to directly infect animals with the SARS‐CoV‐2 and were able to highlight the possible antiviral activity of CBD compounds in a more holistic manner (Figure 1).

The effect of CBD on the clinical evolution of COVID‐19 patients with mild to moderate symptoms was investigated in a clinical trial conducted in Brazil in 2022. During the study, patients received either 300 mg of CBD or placebo added to standard symptomatic therapy for 14 days. As a result, no significant differences could be observed between the COVID‐19 progression of the CBD‐treated and placebo groups. However, it was also suggested by the researchers that increasing the amount of CBD given to patients in future studies could lead to explore any possible therapeutic effect of CBD against COVID‐19.^37^ The National Institute on Drug Abuse has determined that the optimal therapeutic dose of CBD for research is 5 mg; however, the general suggested range is 2.5–10 mg in humans.^38^ In this work, we conducted an experiment using K18‐hACE2 transgenic mice to investigate the antiviral characteristics of CBD. We examined the therapeutic effects of various dosages of CBD, following the dose determination advice by Sea et al. While the effect of CBD was found to be low at low doses and in inappropriate solvents, like corn oil, at the end of the study, we proved how it protects the lungs against the SARS‐CoV‐2 virus when appropriate conditions and relatively high doses are provided (Figure 2D). In 2022, Nguyen and his colleagues conducted a parallel study using transgenic mice with K18‐hACE2 and found that CBD effectively suppressed viral replication in the lungs and nasal concha on the 5th day following infection. This study confirms our findings by providing comparable data.

In another study carried out with cannabichromene (CBC), a type of cannabinoid, the therapeutic effect of CBC on ARDS was investigated. ARDS‐like symptoms were induced as in Salles et al., and a murine model was used for in vivo experiments. Results showed that CBC administration through an inhaler was able to reverse the hypoxia, reduce the pro‐inflammatory cytokines by 50% in the lung and blood, and protect the lung tissues from further destruction.^39^ Besides, in our histopathology study, in parallel with the data obtained using the CBC molecule in the above‐mentioned study, we observed that the lungs of the mice administered CBD were much healthier than the lungs of the control mice that were not treated with CBD (Figure 3A,B).

The antiviral action of CBD, which had been stated in previous studies, was evaluated and proved on mice using the wild SARS‐CoV‐2 virus in this study. Additional experiments to boost CBD efficacy and cytokine studies with animal serum are planned for future research.

## AUTHOR CONTRIBUTIONS

**Hivda Ulbegi Polat:** Data curation (lead); formal analysis (lead); methodology (lead); validation (lead); writing – original draft (lead). **Hicret Asli Yalcin:** Investigation (supporting); writing – original draft (supporting); writing – review and editing (supporting). **Deniz Köm:** Formal analysis (supporting); investigation (supporting); methodology (supporting). **Özge Aksoy:** Formal analysis (supporting); investigation (supporting); methodology (supporting). **Irem Abaci:** Data curation (supporting); investigation (supporting). **Arzu Tas Ekiz:** Data curation (supporting); formal analysis (supporting); methodology (supporting). **Müge Serhatli:** Data curation (supporting); methodology (supporting). **Selma Onarici:** Investigation (lead); project administration (lead); writing – review and editing (lead).

## FUNDING INFORMATION

TUBITAK (The Scientific and Technological Research Council of Türkiye) Marmara Research Center (grant no. 5213301).

## CONFLICT OF INTEREST STATEMENT

The authors declare no conflicts of interest.

## Supporting information


Table S1.

## Data Availability

The data that support the findings of this study are available in the methods and/or supplementary material of this article.
