# Supplementary material for: Antiviral effect of cannabidiol on K18‐hACE2 transgenic mice infected with SARS‐CoV‐2
Source: J Cell Mol Med. 2024 Sep 12;28(17):e70030. doi: 10.1111/jcmm.70030 (PMC11392655; doi:10.1111/jcmm.70030)
Supplement: Supplementary file 1 — Table S1. [file JCMM-28-e70030-s001.docx]

| **Dose-Ranging Studies** | **Groups** | **Animal No** | **Weight change (%)** | **Pulmonar Tissue SARS-CoV-2 Real Time PCR CT** | |
| --- | --- | --- | --- | --- | --- |
|  |  |  |  | **N1** | **N2** |
| **Study 1** | 1µg/mouse CBD in olive oil | **1** | -4,12 | N/A | N/A |
|  |  | **2** | -1,19 | N/A | N/A |
|  |  | 3 | -1,61 | 39 | N/A |
|  |  | **4** | **-15,83** | 15 | 16 |
|  |  | **5** | **-13,18** | 19 | 19 |
|  |  | **6** | 3,49 | N/A | 39 |
|  | Control | **1** | **-25,89** | 14 | 16 |
|  |  | **2** | **-17,81** | 15 | 17 |
|  |  | **3** | **-21,11** | 15 | 15 |
|  |  | **4** | **-23,97** | 17 | 18 |
|  |  | **5** | **-16,35** | 18 | 17 |
|  |  | **6** | **-21,01** | 13 | 14 |
| **Study 2** | **Group 1:** 1µg/ mouse CBD in corn oil | **1** | **-12,84** | 21 | 20 |
|  |  | **2** | **-10,18** | 30 | 28 |
|  |  | **3** | **-25,30** | 24 | 23 |
|  |  | **4** | -3,36 | 28 | 26 |
|  |  | **5** | 1,87 | 35 | 37 |
|  |  | **6** | -6,70 | 33 | 36 |
|  | **Group 2:** 1µg/ mouse CBD in olive oil | **1** | **-13,85** | 38 | 39 |
|  |  | **2** | 0,24 | N/A | N/A |
|  |  | **3** | **-13,22** | 26 | 26 |
|  |  | **4** | -6,43 | N/A | N/A |
|  |  | **5** | -0,52 | N/A | N/A |
|  |  | **6** | 1,32 | N/A | N/A |
|  | **Group 3:** Control | **1** | **-43,97** | 19 | 18 |
|  |  | **2** | **-13,23** | N/A | N/A |
|  |  | **3** | -7,57 | 24 | 23 |
|  |  | **4** | **-20,83** | 25 | 24 |
|  |  | **5** | **-34,81** | 20 | 18 |
|  |  | **6** | -6,91 | 20 | 18 |
| **Study 3** | **Group 1:** 3µg/ mouse CBD in olive oil | **1** | -2,52 | 35 | 36 |
|  |  | **2** | -2,92 | 36 | 35 |
|  |  | **3** | **-9,05** | 32 | 31 |
|  |  | **4** | -0,74 | N/A | N/A |
|  |  | **5** | 4,59 | N/A | N/A |
|  |  | **6** | -2,00 | N/A | N/A |
|  |  | **7** | -1,56 | 35 | 34 |
|  |  | **8** | **-8,95** | 16 | 16 |
|  |  | **9** | 3,24 | N/A | N/A |
|  |  | **10** | **-4,85** | N/A | N/A |
|  | **Group 2:** Control | **1** | **-27,14** | 15 | 16 |
|  |  | **2** | **-26,06** | EX | EX |
|  |  | **3** | -1,11 | N/A | N/A |
|  |  | **4** | **-26,90** | 18 | 18 |
|  |  | **5** | **-23,09** | 16 | 18 |
|  |  | **6** | **-33,74** | 17 | 16 |
|  |  | **7** | **-29,26** | 18 | 18 |
|  |  | **8** | -0,5 | 27 | 27 |
|  |  | **9** | **-28,78** | 16 | 15 |
|  |  | **10** | -1,93 | 25 | 26 |

**Supplementary Table 1** All previous and recent studies on control and experimental group animals; percentages of live weight change and CT values obtained by the N1 and N2 regions of the SARS-CoV-2 virus in real-time PCR.
